# Effect of Aflasafe TZ01^®^ on Aflatoxin Reduction and Emerging Challenges with *Fusarium* Mycotoxins in Maize from Rural Tanzania

**DOI:** 10.3390/toxins17080419

**Published:** 2025-08-19

**Authors:** Sambwe Fundikira, Martin Kimanya, Rashid Suleiman, Marthe De Boevre, Kokeb Tesfamariam, Sarah De Saeger

**Affiliations:** 1 Department of Food Science and Agro-Processing, Sokoine University of Agriculture, Morogoro P.O. Box 3006, Tanzania; rashid@sua.ac.tz; 2 Centre of Excellence in Mycotoxicology and Public Health, Faculty of Pharmaceutical Sciences, Ghent University, Ottergemsesteenweg 460, B-9000 Ghent, Belgium; marthe.deboevre@ugent.be (M.D.B.); kokebtesfamariam.hadush@ugent.be (K.T.); sarah.desaeger@ugent.be (S.D.S.); 3 SACIDS Foundation for One Health, College of Veterinary Medicine and Biomedical Sciences, Sokoine University of Agriculture, Morogoro P.O. Box 3297, Tanzania; 4 School of Life Sciences and Bio-Engineering, The Nelson Mandela African Institution of Science and Technology (NM-AIST), Arusha P.O. Box 447, Tanzania; martin.kimanya@nm-aist.ac.tz; 5 Department of Biotechnology and Food Technology, Faculty of Science, University of Johannesburg, Doornfontein Campus, P.O. Box 17011, Johannesburg 2028, South Africa

**Keywords:** Aflasafe^®^, atoxigenic *Aspergillus flavus*, *Fusarium*, food safety, mycotoxin, Tanzania

## Abstract

Aflatoxins are carcinogenic secondary metabolites produced by *Aspergillus* species and are common contaminants of many crops including maize. Atoxigenic *Aspergillus flavus* strains, formulated as biocontrol products such as Aflasafe^®^ TZ01, that comprises a mixture of four native atoxigenic strains, are used as pre-harvest agents to suppress toxigenic strains and reduce aflatoxin levels. This study assessed the intended and potential unintended impacts of Aflasafe^®^ TZ01 on mycotoxin contamination in maize. A total of 158 samples 79 from treated and 79 from untreated fields were collected from Chemba and Kiteto districts in Tanzania. Multi-mycotoxin analysis was conducted using liquid chromatography coupled with tandem mass spectrometry (LC-MS/MS). Detected toxins included aflatoxins (AFB1, AFB2, AFG1, AFG2), trichothecenes, and fumonisins (FB1, FB2, FB3). Non-parametric paired t-test analysis showed significant reductions in AFB1 (62%, *p* = 0.024) in treated samples. The mean concentrations of *Fusarium* mycotoxins such as NIV, T2, and ZEN were higher in treated maize. However, statistical analysis showed that these differences were only numerical trends, and were not significant (*p* > 0.05). These findings confirm the efficacy of Aflasafe^®^ TZ01 in reducing aflatoxins, while underscoring the importance of continued monitoring for other mycotoxins as part of integrated mycotoxin management strategies to mitigate both aflatoxins and co-occurring toxins.

## 1. Introduction

Maize (*Zea mays* L.) is a vital cereal crop across sub-Saharan Africa, particularly as a staple food and a key source of income for millions of smallholder farmers [1,2]. In Tanzania, maize plays a central role in both rural and urban diets, with an annual per capita consumption reaching approximately 128 kg, making it a primary food source [3]. However, like other staple crops such as sorghum, groundnuts, and millet, maize is highly vulnerable to fungal infestation and consequent contamination by mycotoxins, especially aflatoxins and fumonisins, which pose significant health risks [4].

Aflatoxins, primarily produced by *Aspergillus flavus* and *A. parasiticus*, are among the most harmful mycotoxins affecting maize [5]. These toxins are considered a serious public health concern especially in sub-Saharan Africa, where they significantly affect food safety, economical and agricultural productivity [6]. Aflatoxin B1 (AFB1), the most potent of these toxins, is classified as a Group 1 carcinogen by the International Agency for Research on Cancer (IARC), with its long-term exposure linked to liver cancer, immune suppression, and stunted growth in children [7,8,9,10]. Aflatoxin contamination leads to mycotoxicosis, which can be acute or chronic, with the latter resulting from prolonged exposure to small amounts of toxins over time [11,12] and the former being the exposure of high dose of the toxin at one point. Aflatoxin outbreaks have been documented in sub-Saharan Africa, including the incidences of 2016 and 2019 in Tanzania’s Chemba and Kiteto districts, where causalities and deaths were reported after consuming maize-related foods [12,13]. Other studies in Tanzania have revealed a high prevalence of aflatoxin contamination (10–80%) in maize, with a mean concentration ranging from 12.47 to 162.40 μg/kg, indicating a high risk of human exposure to aflatoxin due to the high average maize consumption rate per day per person [14].

In addition to aflatoxins, maize is increasingly contaminated by *Fusarium*-derived mycotoxins such as fumonisins, deoxynivalenol (DON), and nivalenol (NIV) [15]. Recent findings indicate co-occurrence of multiple mycotoxins, raising concerns about synergistic toxicity [16,17]. A study conducted by Kamala et al. [16] revealed a high level of AFs and FBs in 45% of the 120 samples analyzed, which were collected from different households in Tanzania. Another study showed that fumonisins were detected in 52% of the samples at a maximum level of 11,048 μg/kg [17]. Moreover, the study found 15% of the contaminated samples exceeded the maximum limits of 1000 μg/kg set by European regulations (EU) 2023/915 [17]. At the national level, regulations for maize in Tanzania focus only on aflatoxins and fumonisins, while overlooking other harmful mycotoxins like zearalenone, deoxynivalenol, T-2, and ochratoxin A, among other mycotoxins which also pose health risk to the public [15].

Within the *Aspergillus* genus, only certain strains possess the genetic capacity to synthesize aflatoxins, while others, termed non-aflatoxigenic, lack functional genes for aflatoxin biosynthesis and therefore do not produce these toxins [18]. Among these species, aflatoxigenic *A. flavus* is the most prevalent in crops that are prone to aflatoxin and cyclopiazonic acid contamination, whereas non-aflatoxigenic strains are rare [19,20]. *A. flavus* naturally exists as a saprophyte in the soil playing an important role in decomposition of organic matter and in nutrient biogeochemical cycles. Under certain circumstances *A. flavus* can attack crops in the field, exists as an opportunistic parasite and causes diseases such as ear rot in maize and yellow mold in peanuts [15]. When crops are stressed due to environmental conditions (e.g., drought) or biotic conditions (e.g., pest and disease infestations), they become susceptible to *Aspergillus* proliferation, and thus contamination with mycotoxins [21].

Several studies have been conducted worldwide to demonstrate the efficacy and safety of the use of atoxigenic agents, including the study conducted by Grubisha and Cotty in 2015 in the United States which confirmed the genetic stability of biocontrol strain AF36 [22]. Additional population genetic studies from Africa and Europe corroborate these findings, showing genetic stability of biocontrol strains under diverse agroecological conditions [23,24,25]. These findings support the conclusion that atoxigenic biocontrol strains maintain their intended function without genetic reversion or unintended ecological impacts over multiple seasons.

To combat aflatoxin contamination, Tanzania approved the use of the biocontrol agent Aflasafe^®^ TZ01 in 2018 [26]. This product contains native, non-aflatoxigenic strains of *A. flavus* that competitively exclude toxigenic strains, thereby reducing aflatoxin levels in crops [27]. Biocontrol heavily relies on screening and natural selection. Careful consideration should be and is normally given during selection to ensure that biocontrol agents possess the necessary characteristics to achieve optimal results in the tested strains [28]. Therefore, the inappropriate selection of atoxigenic strains may inadvertently result in the selection of strains capable of producing aflatoxins and other metabolites such as cyclopiazonic acid, which is concurrently produced by certain *A. flavus* strains [29].

Despite these successes, other researcher have questioned the possibility of atoxigenic *Aspergillus* to create other toxins production while reducing aflatoxins [30,31,32]. In addition, a study conducted in Ghana and Nigeria to check if atoxigenic biocontrol increase fumonisins content found one case where fumonisins content was significantly higher in treated fields in Upper East Ghana [33]. Questions remain regarding the broader ecological effects of this biocontrol intervention, particularly its influence on co-occurring fungi such as *Fusarium* spp. and the potential production of other mycotoxins [31]. Moreover, the capacity of non-aflatoxigenic strains to acquire toxigenic traits through recombination is not fully understood, highlighting the need for careful strain selection and monitoring [29].

Furthermore, while biocontrol strategies have consistently reduced aflatoxin contamination, limited information exists on their impact on the presence and concentrations of other mycotoxins. Interactions between atoxigenic and toxigenic strains, as well as their potential influence on mycotoxins like fumonisins and trichothecenes produced by *Fusarium* spp., represent significant gaps. In Tanzanian farming systems, the effect of such technology on other fungi remains largely unexplored.

This study aims to assess the effect of Aflasafe^®^ TZ01 application on the occurrence of multiple mycotoxins in maize harvested from Chemba and Kiteto districts in Tanzania, thereby contributing to a more holistic understanding of biocontrol efficacy and safety.

## 2. Results

### 2.1. Method Performance Criteria

Table 1 represents the method validation parameters for the multi-mycotoxin analysis conducted on maize samples. The parameters reported include the coefficient of determination (R^2^), apparent mean recovery with standard deviation, limit of detection (LOD), and limit of quantification (LOQ) for each mycotoxin. The method demonstrates excellent linearity for most mycotoxins, with R^2^ values generally above 0.99, indicating a strong linear correlation between the analyte concentration and instrument response. This high level of linearity is crucial for accurate quantification across a range of concentrations [34]. Apparent mean recoveries for all mycotoxins fall within the range of 95% to 110%, with most clustered around 100–105%. These recovery rates are well within the acceptable range of 70–120% recommended by the European Commission Implementing Regulation (EU) No 2023/2782 for methods of sampling and analysis for the control of the levels of mycotoxins in food.

The limits of detection (LOD) and quantification (LOQ) varied considerably among the different mycotoxins, reflecting differences in their chemical properties and instrumental responses. For instance, aflatoxins (AFG2, AFG1, AFB2, AFB1) show low LOD and LOQ values (0.08–0.32 µg/kg and 0.23–0.98 µg/kg, respectively), which is crucial given their high toxicity and strict regulatory limits [35].

### 2.2. Treatment Effects on Mycotoxin Accumulation in Maize Samples

The mean concentrations and ranges of mycotoxin levels in treated and control fields maize samples were compared to assess the effectiveness of Aflasafe TZ01^®^ in reducing contamination (Table 2). For the details of the detected mycotoxins concentration, see Supplementary Table S2.

### 2.3. Anticipated Effect of Aflasafe^®^ TZ01

Aflatoxin concentrations were significantly reduced in maize treated with Aflasafe^®^ TZ01 compared to the untreated control. The average concentration of AFB1 in the treatment group was 0.73 µg/kg, compared to 1.86 µg/kg in the control, representing a 61% reduction. Similarly, AFG1 levels decreased from 1.71 µg/kg in the control to 0.14 µg/kg in the treatment group a 92% reduction. Likewise, AFG2 levels were also reduced from 0.32 µg/kg in the control to 0.12 µg/kg following treatment, reflecting a 63% decrease. Also there was insignificant reduction of fumonisins with lower percentages (10–20%). To ensure statistical reliability, mycotoxins detected in fewer than 10% samples in both control and treatment were excluded from statistical analysis.

### 2.4. Post-Treatment Trends in Fusarium-Produced Mycotoxins

While numerical increases in certain *Fusarium*-associated mycotoxins were observed following treatment, these differences were not statistically significant. Specifically, the mean concentration of nivalenol (NIV) increased from 6.40 µg/kg in the control group to 10.01 µg/kg in the treatment group (a 55.4% increase), and T-2 toxin levels rose slightly from 0.06 µg/kg to 0.10 µg/kg (58.71% increase). Zearalenone (ZEN), although showing a large numerical rise from 1.04 µg/kg to 13.80 µg/kg (1226.61% increase), also exhibited high variability among samples and did not reach statistical significance. Likewise, sterigmatocystin (STE) showed a modest increase from 0.09 µg/kg in the control to 0.11 µg/kg in the treatment group (15.32% increase), which was also not significant. These results reveal the observed effects of biocontrol on different mycotoxins. To quantify these effects, percentage reduction was calculated only to the detectable mycotoxins.

### 2.5. Biocontrol Efficacy on Mycotoxins Levels: A Mixed Impact

Table 3 presents the *p*-values indicating the statistical significance of the effects of biocontrol treatment on different mycotoxins in maize samples from Kiteto and Chemba districts. The effect of biocontrol treatment on mycotoxin concentrations showed both positive and negative outcomes as explained above highlighting its complex interaction with fungal communities. Statistically significant reduction (*p* < 0.05) was observed for AFB1 indicating that biocontrol effectively suppressed toxigenic *Aspergillus*, leading to lower contamination level. These results demonstrate the effectiveness of Aflasafe^®^ TZ01 in reducing aflatoxins B1 contamination and highlight areas where additional management strategies may be necessary. Also, mycotoxins detected in fewer than 10% samples in both control and treatment were excluded from statistical analysis.

### 2.6. District Variation in Biocontrol Efficacy

Further statistical analysis was performed to determine if there were significant differences between the locations (Chemba and Kiteto) in terms of biocontrol efficacy against various mycotoxins by using a t-test. In Chemba, the biocontrol treatment significantly reduced the concentrations of aflatoxins, including AFG1 (*p* = 0.0564) and AFB1 (*p* = 0.0200), indicating a strong suppressive effect on aflatoxin-producing fungi, likely *Aspergillus flavus* [36]. Additionally, OTA (*p* = 0.0389), and STE (*p* = 0.0332) were significantly reduced, showing the potential of biocontrol in controlling both aflatoxins and certain *Penicillium*-related mycotoxins. Highly significant reductions were observed for AME (at *p* < 0.01), indicating an especially strong biocontrol effect on these mycotoxins.

In contrast, the biocontrol treatment in Kiteto did not show any significant reduction for aflatoxins such as AFB1 (*p* = 0.5638) and AFG1 (*p* = 0.6061), suggesting that the biocontrol agent might have had limited effectiveness against *Aspergillus* species in this region. This might be due to suboptimal moisture availability and high temperatures, reducing its competitive advantage.

## 3. Discussion

### 3.1. Aflatoxins and Fumonisins Incidence Above Regulatory Levels

AFB1 contamination in the analyzed samples ranged from 0.32 to 76 µg/kg, with more than 2.5% exceeding the Tanzania standard (TZS 438)/East African Standards (EAS 2) regulatory limit of 5 µg/kg [37]. Additionally, 3.2% of the total 158 samples surpassed the EAS 2/TZS 438 maximum allowable limit for total aflatoxins of 10 µg/kg, with the highest concentration recorded at 90.58 µg/kg. Regarding fumonisins, over 15% of the samples exceeded the EAS regulatory limit of 2000 µg/kg, with concentrations ranging from 1.35 to 14,117.93 µg/kg. However, when assessed against the Codex Alimentarius limit of 4000 µg/kg, only 4% of the samples were non-compliant.

### 3.2. Effectiveness of Aflasafe^®^ TZ01 Biocontrol in Reducing Aflatoxin Contamination in Maize

This study was conducted in Kiteto and Chemba districts of Tanzania aimed to evaluate the impact of the Aflasafe^®^ TZ01 biocontrol application on mycotoxin contamination in maize. The results revealed substantial reductions in aflatoxin levels, with AFB1, AFB2, AFG1, and AFG2 showing mean reductions of 62%, 100%, 93%, and 63% respectively. Among these, only the reduction of AFB1 was statistically significant. These results demonstrate the effectiveness of Aflasafe^®^ TZ01 in mitigating aflatoxin levels under field conditions. The significant decrease in aflatoxin levels underscores the potential of biocontrol agents as a viable pre-harvest intervention for enhancing food safety. These findings are consistent with previous studies revealing the effectiveness of Aflasafe^®^ TZ01 in reducing aflatoxin contamination up to 90% by outcompeting toxigenic *Aspergillus flavus* strains in the field [7,23,24,38,39,40].

### 3.3. Unintended Consequences of Aflasafe^®^ TZ01 Application on Fusarium Toxins

The study revealed notable increases in *Fusarium* toxins concentrations specifically, NIV, T2, ZEN, and the *Aspergillus* toxin STE and OTA. Aflasafe^®^ TZ01 works on the principle of competitive exclusion, introducing non-toxigenic strains of *Aspergillus flavus* to outcompete toxigenic strains. However, this may inadvertently create an ecological niche for other fungi, including *Fusarium* species. Other researchers in their review [4] of biocontrol strategies for aflatoxin mitigation, highlighted that although such approaches are effective against targeted aflatoxigenic species, they may have limited efficacy in controlling other fungi responsible for producing different types mycotoxin. The detection of *Fusarium* mycotoxins in a small proportion of maize samples from Aflasafe^®^ TZ01 treated fields is consistent with previous studies showing that atoxigenic *Aspergillus flavus* biocontrol products are specifically developed to reduce aflatoxin contamination, but do not directly impact the occurrence or production of toxins by *Fusarium* species [4,31,33,41]. The presence of *Fusarium* toxins in both treated and untreated fields likely reflects the influence of environmental factors, maize genotype, pest pressure, and agronomic practices, rather than any unintended effect of the biocontrol intervention itself [33]. The observed insignificant results on the increase of *Fusarium* toxins in Aflasafe^®^ TZ01 treated fields, is similar to the study conducted in Italy where higher occurrences were also noted in treated fields, and the differences between control and treatment were not statistically significant [41].

In contrast to our results, Reis et al. [42] found that the use of atoxigenic *Aspergillus* strains led to a reduction in the prevalence of *Fusarium verticillioides* and a corresponding decrease in fumonisin levels.

Biocontrol with atoxigenic *Aspergillus flavus* strains selectively targets and suppresses toxigenic *A. flavus* populations by promoting the establishment and competitive dominance of atoxigenic strains, thereby reducing aflatoxin contamination while maintaining the overall *Aspergillus* community structure [23,25,43]. However, as toxigenic *A. flavus* populations are suppressed, *Fusarium* species may face reduced competition for nutrients and colonization sites, potentially leading to their increased proliferation and heightened production of toxins such as fumonisins and trichothecenes [30,31,44,45]. In addition, the tropical climatic conditions in Kiteto and Chemba support both *Aspergillus* and *Fusarium* growth. The suppression of *Aspergillus* by Aflasafe^®^ TZ01 may allow *Fusarium* species to better exploit the environmental conditions [46]. Different mycotoxin-producing fungi can thrive under varied environmental conditions in Tanzania, indicating complex interactions between fungal species, environmental factors, crops and management practices [16]. Moreover, other researchers have explained the presence of non-toxigenic *Aspergillus* strains introduced by Aflasafe^®^ TZ01 may influence the metabolism of *Fusarium* species through indirect interactions. This could potentially lead to increased production of certain mycotoxins as a stress response or competitive mechanism. The researcher proposed that changes in one segment of the microbial ecosystem, such as the suppression of *Aspergillus* by Aflasafe^®^ TZ01, could lead to niche expansion for various *Fusarium* species with different mycotoxin profiles [47]. Although studies assessing the impact of Aflasafe^®^ TZ01^®^ on non-aflatoxin mycotoxins remain limited, one study conducted in Canada evaluated the effects of *Clonostachys rosea*, a biocontrol agent targeting *Fusarium graminearum*, on mycotoxin production in wheat [48]. They found that while the biocontrol agent effectively reduced deoxynivalenol (DON) levels, it also led to increase in production of nivalenol (NIV) in some *F. graminearum* chemotypes. This finding parallels our observation of shifts in mycotoxin profiles, suggesting that biocontrol interventions can have complex effects on fungal communities and their metabolic outputs.

### 3.4. Challenges of Mycotoxin Co-Occurrence and Implications for Risk Management

The analysis of both treated and control maize samples (158 samples) revealed a significant co-occurrence of multiple mycotoxins, particularly fumonisins, trichothecenes, and aflatoxins. Among the detected mycotoxins, fumonisins (FB1, FB2, and FB3) were the most prevalent, appearing in over 80% of samples in both treated and control groups. The co-occurrence of these mycotoxins is particularly concerning due to potential synergistic effects. As noted by Smith et al. [49] the combined presence of multiple mycotoxins can lead to additive or even synergistic toxicological effects, potentially increasing the overall health risk to humans and animals consuming contaminated crops [50,51]. For instance, Alassane-Kpembi et al. [52] demonstrated that combinations of different trichothecenes, including DON and its acetylated derivatives, can have synergistic cytotoxic effects at low doses.

Moreover, the co-occurrence of these mycotoxins presents challenges for regulatory compliance and food safety. As [53] discussed, most regulatory limits focus on individual mycotoxins, but the reality of multiple mycotoxin contamination calls for more comprehensive approaches to risk assessment and management. The regulation of mycotoxins in East Africa only focuses on aflatoxins and fumonisins while overlooking other toxic mycotoxins like DON and ZEN which also co-exist in maize [15]. This calls for a review of our regulatory standards for maize and other cereals to include the unregulated mycotoxins which also pose a threat to public health.

This study underscores the necessity for integrated pest management strategies that address multiple mycotoxin-producing fungi. For instance, combining biocontrol agents like Aflasafe^®^ TZ01 with improved agricultural practices and better storage facilities could mitigate the risk of contamination by both *Aspergillus* and *Fusarium* species [54]. As highlighted by [55], good agricultural practices (GAP) serve as the primary defense against mycotoxin contamination in food crops, both during cultivation and post-harvest. To further ensure food safety, these practices should be reinforced by good manufacturing practices (GMP) throughout processing, storage, and distribution, minimizing contamination risks along the supply chain. Additionally, ongoing education and training for farmers on mycotoxin management are crucial for sustaining the benefits of biocontrol interventions [56].

### 3.5. Limitations of the Study

This study did not analyze the soil fungal community. While this study provides important insights into the occurrence of multiple mycotoxins in maize from fields treated with Aflasafe^®^ TZ01, it is important to acknowledge that the absence of fungal community analysis limits our ability to establish causal relationships. The associations observed in this study should be interpreted as correlative rather than causal. We recommend that future studies integrate fungal community profiling techniques to unravel the ecological interactions between *Aspergillus* and *Fusarium* species and better understand the broader impacts of biocontrol strategies under field conditions.

Also the small number of contaminated samples and absence of statistical significance are a limitation to drawing broader conclusions about unintended effects.

## 4. Conclusions

In conclusion, our study investigated the effect of Aflasafe^®^ TZ01 on aflatoxins and other mycotoxins. While Aflasafe^®^ TZ01 significantly reduced aflatoxin contamination in maize, the observed increase in *Fusarium* mycotoxins highlights the complexity of mycotoxin management in tropical climates. Despite this increase, the proportion of positive samples between treatment and control fields is small, suggesting that the biocontrol treatment influenced toxin intensity rather than prevalence. These findings emphasize the need for holistic approaches that incorporate environmental, agricultural, and storage practices to ensure comprehensive food safety in regions like Kiteto and Chemba [4,55].

Our findings serve as preliminary, reflecting the limited incidence and absence of significant change of increased *Fusarium* toxins following Aflasafe^®^ TZ01^®^ application across the dataset. Further research is warranted to elucidate the mechanisms driving these shifts in mycotoxin profiles, explore the study in multiple years/ locations and to develop integrated management approaches that can effectively address multiple mycotoxin risks simultaneously. This involves combining biocontrol strategies with other management practices such as resistant maize varieties, pest control, and improved post-harvest handling or developing multi-species biocontrol approaches that target a broader range of mycotoxin-producing fungi.

## 5. Materials and Methods

### 5.1. Study Area

The study was carried out in two different districts (Chemba in Dodoma Region, and Kiteto in Manyara Region) of Tanzania in the 2021–2022 growing season. The districts are characterized by a semi-arid climate with marked seasonal variations in temperature and rainfall. The districts receive annual rainfall between 400 mm and 800 mm, with the main rainy season occurring from November to April, followed by a dry season. The average temperatures range from 15 °C to 31 °C with higher temperatures typically occurring in the dry season (https://weatherspark.com/y/99536/Average-Weather-in-Kibaya-Tanzania-Year-Round#) (accessed on 10 May 2024). These conditions create an environment favorable for the growth of *Aspergillus* spp. and *Fusarium* spp. which thrive in hot and humid conditions, particularly, when crops are exposed to moisture stress followed by sudden rains. The selection criteria of the study sites (districts) were based on the previous reports on aflatoxicosis outbreaks which were caused by the consumption of contaminated maize [12,13]. Additionally, the selected districts are among the major maize producing regions in the country, making them critical for assessing both exposure risk and food safety concerns related to mycotoxin contamination.

### 5.2. Participants and Experimental Design

Participants in the study were selected from a list of farmers obtained from the agriculture extension officers in charge in each district. Farmers with plot sizes not exceeding two acres growing maize mainly for home consumption were purposively selected to participate in this study. Efficacy assessment of biocontrol in reducing *Aspergillus* secondary metabolites was verified by farmers who gave their consent to take part in the study. Briefly, in each village, farmers were divided into two groups (treatment and control) with a minimum of 0.1 km separation between farmers with treated and farmers with corresponding control fields to avoid cross-contamination of the biocontrol isolates [40]. Both treatment and control fields represented the same climatic condition and soil characteristics as they fall in the same agro-ecological zone. Farmers were advised to follow good agriculture practices (GAP) for both treatment and control fields without any special intervention. Samples of maize were collected from the treatment and control groups (soon after harvest). The distribution of samples is as shown in Table 4.

### 5.3. Aflasafe^®^ TZ01 Application and Initial Sample Preparation

The biocontrol product, Aflasafe TZ01^®^ manufactured by A to Z Textile Mills Limited in Arusha, Tanzania, contains atoxigenic strains of *Aspergillus flavus* indigenous to Tanzania was supplied to farmers at the time of application. The application of Aflasafe^®^ TZ01 was done by hand broadcast on the soil surface of the maize plots, 2–3 weeks before flowering as elaborated by [43]. The experimental fields size ranged from 0.25 to 1 acre. The protocol for the application of biocontrol material was demonstrated by agricultural extension officers, in the farmers’ fields. Application of Aflasafe^®^ TZ01 was done when the soil was wet to allow maximum sporulation of the atoxigenic strains. Farmers were advised to finalize agronomic practices before application of the product and reduce movement on the farm for 7 to 10 days after biocontrol application to avoid burying of the product [38].

At harvest, five quadrants of approximately 5 m × 5 m were identified in each experimental field (treatment vs. control) whereby five cobs were selected and picked, resulting in 25 cobs per field. Dried maize samples in cobs were collected during harvest and stored in airtight polyethylene bags and, in 2 days, transported to Sokoine University, Food science and Agro-processing laboratory for further preparation. In the laboratory the maize cobs were dehulled by using a manual huller. Further drying by air oven at a temperature of 105 °C ± 5 °C for 12 h was done to the moisture content of 14% m/m. Then, 2 kg of the dried sample were grinded and a 50 g subsample was stored in a sealed zip lock bag and stored in a freezer at −20 °C at the Food Science and Agro-Processing Laboratory for further analysis at the Centre of Excellence in Mycotoxicology at Ghent University, Belgium.

### 5.4. Quality Control of the Multi-Mycotoxins Methodology

Analysis started with creation of a calibration curve by spiking blank maize samples with recognized reference standards, in accordance with the guidelines of EN ISO 17025. De-epoxy-deoxynivalenol (DOM) and zearalanone (ZAN) were employed as internal standards in the analysis of multi-mycotoxins in maize samples. These standards were chosen for their structural similarity to target mycotoxins, ensuring they exhibit similar behavior during extraction, separation, and mass detection processes. This similarity allows accurate compensation for variability in analytical procedures, thereby enhancing the precision and accuracy of the quantification [57]. Moreover, DOM and ZAN are stable molecules and not naturally present in food samples, preventing interference with the target analytes [58]. The limits of detection (LOD) and quantitation (LOQ) were established at 3.33 and 10 times the signal-to-noise ratio, respectively to determine the sensitivity of the method. Quantitation was carried out by constructing matrix-matched calibration plots using the least squares method. The plots were created by plotting the relative peak area (the ratio of the mycotoxins response to the corresponding peak area of the internal standard) against the spiked concentrations. This approach ensured accuracy and precision in the quantification process.

Additionally the presence of the compound could only be confirmed if four criteria were simultaneously fulfilled as described under the EU Commission Decision, 2002/657/EC, the Directive EC 2002/Implementing Council Directive 96/23/EC and SANTE guidance document on identification of mycotoxins and plant toxins in food and feed concerning the performance of analytical methods and the interpretation of results.

For accurate analysis, several key criteria must be met. At least two selected fragment ions must be present simultaneously at the same retention time. These fragment ions must demonstrate a signal-to-noise ratio exceeding 3 to ensure reliable detection. Likewise, the component’s relative retention time in relation to the internal standard must fall within a ±2.5% margin. Furthermore, when analyzing the relative intensity of the selected ions (expressed as a percentage of the most abundant ion’s intensity), these values must align with the ion intensities observed at the cut-off calibration point for quantitative analysis.

Method performance criteria are as elaborated in Table 1.

### 5.5. Extraction, Purification, and Evaporation

From the representative ground maize samples, a total of 3 g from both treatment and control fields were accurately weighed and placed into 50 mL extraction tubes. Subsequently, 20 mL of extraction solvent (methanol/ethyl acetate/water of 70/20/10, *v*/*v*/*v*) were added to each tube and thoroughly mixed using a vortex mixer for 1 min. The resulting mixture was then agitated (Exacta, Mery-sur-Oise, France) for 40 min and subsequently centrifuged for 10 min at a speed of 4000 rpm. Following centrifugation, the supernatants were carefully transferred to new extraction tubes, and 10 mL of n-hexane were added to each tube. The tubes were then agitated for 15 min and centrifuged for an additional 15 min at 4000 rpm. The resulting hexane layer was separated, and the sample extract was divided into two parts for liquid–liquid extraction (LLE) and purification using the Amino SPE column (Phenomenex, Utrecht, The Netherlands). For LLE, 2.5 mL of the defatted sample extract were transferred into the extraction tube of 50 mL containing 10 mL dichloromethane/formic acid solution (95/5, *v*/*v*), vortexed and centrifuged at 4000× *g* for 10 min. The remaining fraction of the defatted extract was applied onto the amino SPE column which was prior conditioned by extraction solvent of methanol/ethyl acetate/water (70/20/10, *v*/*v*/*v*) and the eluate was collected in a test tube. The combined eluate (SPE purification and LLE) was evaporated at 600 °C under gentle supply of nitrogen flow. The resulting residue was then re-dissolved using 300 µL of injection solvent and thoroughly mixed using a vortex mixer. Subsequently, 200 µL of n-hexane were added to the mixture, which was then vortexed and centrifuged for 5 min at a speed of 3300× *g*. The dissolved residue was then transferred into centrifuge filters (Merck Millipore, Tullagreen, Carrigtwohill, Ireland) and centrifuged once more. Finally, 100 µL of the resulting filtrate were carefully transferred into vials containing 100 µL injection solvent (water/methanol/acetic acid (2/97/1, *v*/*v*/*v*) + 5 mM ammonium acetate and water/methanol/acetic acid (94/5/1, *v*/*v*/*v*) + 5 mM ammonium acetate in the ratio of 1/1) which were then prepared for LC-MS/MS analysis.

### 5.6. High Performance Liquid Chromatography-Tandem Mass Spectrometry

The identification and quantification of mycotoxins were conducted using high performance liquid chromatography–tandem mass spectrometry (HPLC-MS/MS). The analysis was performed with a Waters Acquity HPLC system connected to a Quattro Premier Tandem Mass Spectrometer equipped with MassLynx^®^ (Waters, Milford, MA, USA). The system utilized a C18 column with an internal diameter of 5 µm and dimensions of 150 mm × 2.1 mm, along with a guard column of similar properties (2.1 mm × 10 mm) (Waters, Zellik, Belgium). Each sample was injected with a volume of 10 µL. The mobile phases (water/methanol/acetic acid (94/5/1, *v*/*v*/*v*) + 5 mM ammonium acetate and water/methanol/acetic acid (2/97/1, *v*/*v*/*v*) + 5 mM ammonium acetate) were set at a flow rate of 0.3 mL/min, and the total run time per sample was 28 min. Instrument control and data processing were managed using Masslynx and Quanlynx software version 4.1 (Manchester, UK) to quantify 22 different mycotoxins. Details of MS/MS parameters is as described in Supplementary Table S1).

The mycotoxins analyzed included aflatoxins (AFB1, AFB2, AFG1, AFG2), nivalenol (NIV), deoxynivalenol (DON), neosolaniol (NEO), 3-acetyldeoxynivalenol (3-AcDON), 15-acetyldeoxynivalenol (15-AcDON), fumonisins (FB1, FB2, FB3), T2-toxin (T2), HT2-toxin (HT2), fusarenon-X (FUS-X), ochratoxin A (OTA), zearalenone (ZEN), diacetoxyscirpenol (DAS), roquefortine-C (ROQ-C), sterigmatocystin (STE), alternariol monomethyl ether (AME), alternariol (AOH), neosolaniol (NEO), and sterigmatocystin (STE).

### 5.7. Statistical Analysis

The normality of distribution of mycotoxin contamination data was assessed using the Shapiro–Wilk test, which indicated a deviation from normality [59]. As a result, the Wilcoxon signed-rank test for censored data, a non-parametric alternative to the paired t-test, was employed to compare median mycotoxin concentrations between control and treatment groups. This test was chosen for its suitability in analyzing non-normally distributed data and its robustness when comparing two related samples. To address left-censored data, a conservative middle-bound substitution method was applied, where mycotoxin concentrations below the limit of detection (LOD) were replaced with one-half the LOD value [59]. This approach is widely used to estimate non-detects while minimizing potential bias in statistical interpretation (https://www.hbm4eu.eu/wp-content/uploads/2018/09/Deliverable-10.2-Statistical-Analysis-Plan.pdf) (accessed on 15 July 2025). All analyses were conducted using Stata Statistical Software (version 17; StataCorp LLC, College Station, TX, USA), with two-sided statistical significance set at *p* < 0.05. Also, to check the treatment effect as the primary objective, we calculated mean reduction of mycotoxins comparing the two groups (treatment and control).

## Figures and Tables

**Table 1:** Performance parameters of mycotoxin analysis.

Mycotoxins	R^2^	Apparent Mean Recovery (%)	LOD (µg/kg)	LOQ (µg/kg)
AFG2	0.9972	102 ± 7.4	0.08	0.23
AFG1	0.9961	100 ± 9.9	0.19	0.59
AFB2	0.9943	102 ± 6.0	0.14	0.41
AFB1	0.9976	100 ± 5.6	0.32	0.98
OTA	0.9809	95 ± 19	1.27	3.86
FB1	0.9943	99 ± 5.7	3.66	11.1
FB2	0.9982	99 ± 3.1	1.35	4.09
FB3	0.9936	98 ± 6.3	3.47	10.5
DON	0.9664	105 ± 23	2.74	8.29
3-AcDON	0.9923	103 ± 8.3	2.36	7.17
15-AcDON	0.9975	101 ± 5.0	14.1	42.7
T2	0.9950	103 ± 7.6	0.17	0.50
HT2	0.9944	102 ± 10	0.94	2.84
DAS	0.9979	102 ± 6.1	0.13	0.38
NIV	0.9733	108 ± 26	6.34	19.2
ZEN	0.9955	104 ± 14	1.70	5.16
FUS-X	0.9956	103 ± 9.1	1.72	5.24
NEO	0.9982	102 ± 8.4	0.39	1.21
STE	0.9946	96 ± 14	0.22	0.67
AME	0.9818	104 ± 11	6.27	19.0
AOH	0.9933	102 ± 6.3	0.79	2.42
ROQ-C	0.9534	110 ± 27	1.38	4.17

Limit of detection (LOD), limit of quantification (LOQ), coefficient of determination (R^2^), aflatoxin B1 (AFB1), aflatoxin B2 (AFB2), aflatoxin G1 (AFG1), aflatoxin G2 (AFG2), ochratoxin A (OTA), fumonisin B1 (FB1), fumonisin B2 (FB2), fumonisin B3 (FB3), deoxynivalenol (DON), 3-acetyldeoxynivalenol (3-AcDON), 15-acetyldeoxynivalenol (15-AcDON), T-2 toxin (T2), HT-2 toxin (HT2), diacetoxyscirpenol (DAS),nivalenol (NIV), zearalenone (ZEN), fusarenon-X (FUS-X), neosolaniol (NEO), sterigmatocystin (STE), alternariol monomethyl ether (AME), alternariol (AOH), and roquefortine-C (ROQ-C).

**Table 2:** Multi-mycotoxin concentrations in maize of both treatment and control fields in Chemba and Kiteto districts in Tanzania.

Mycotoxins	Treated (T)		Control (C)	
	Positive Samples, n (%)	Range (µg/kg)	Positive Samples, n (%)	Range (µg/kg)
AFB1	2 (3%)	˂LOD–54.25	10 (13%)	˂LOD–76.01
AFB2	-	-	5 (6%)	˂LOD–10.91
AFG1	3 (4%)	˂LOD–5.92	9 (11%)	˂LOD–51.92
AFG2	18 (23%)	˂LOD–5.68	24 (30%)	˂LOD–6.36
OTA	11 (14%)	˂LOD–14.46	9 (11%)	˂LOD–13.52
FB1	63 (80%)	˂LOD–5007.17	64 (81%)	˂LOD–10,649.76
FB2	55 (70%)	˂LOD–1022.27	62 (78%)	˂LOD–2765.21
FB3	52 (66%)	˂LOD–854.42	54 (68%)	˂LOD–942.56
DON	4 (5%)	˂LOD–1676.73	2 (3%)	˂LOD–151.51
3-AcDON	2 (3%)	˂LOD–93.41	1 (1%)	˂LOD–15.77
15-AcDON	3 (4%)	˂LOD–338.08	1 (1%)	˂LOD–97.72
T2	6 (8%)	˂LOD–5.44	12 (15%)	˂LOD–0.48
DAS	6 (8%)	˂LOD–24.94	6 (8%)	˂LOD–1.42
NIV	26 (33%)	˂LOD–179.90	24 (30%)	˂LOD–59.70
ZEN	11 (14%)	˂LOD–782.14	14 (18%)	˂LOD–14.81
FUS-X	29 (37%)	˂LOD–61.27	36 (46%)	˂LOD–58.83
NEO	2 (3%)	˂LOD–130.16	-	-
STE	4 (5%)	˂LOD–6.53	11 (14%)	˂LOD–1.20
AME	ND	ND	6 (8%)	˂LOD–22.78
AOH	ND	ND	2 (3%)	˂LOD–1.13

Treated—with Aflasafe^®^ TZ01 (Atoxigenic strains of *Aspergillus flavus*); Control—not with Aflasafe^®^ TZ01 (Atoxigenic strains of *Aspergillus flavus*); Positive samples are those with values above the limit of detection (LOD), with ‘n’ representing positive samples analyzed per group and % calculated on 79 total samples per group. Aflatoxin B1 (AFB1), aflatoxin B2 (AFB2), aflatoxin G1 (AFG1), aflatoxin G2 (AFG2), ochratoxin A (OTA), fumonisin B1 (FB1), fumonisin B2 (FB2), fumonisin B3 (FB3), deoxynivalenol (DON), 3-acetyldeoxynivalenol (3-AcDON), 15-acetyldeoxynivalenol (15-AcDON), T-2 toxin (T2), diacetoxyscirpenol (DAS),nivalenol (NIV), zearalenone (ZEN), fusarenon-X (FUS-X), neosolaniol (NEO), sterigmatocystin (STE), alternariol monomethyl ether (AME), and alternariol (AOH); “ND” = mycotoxin was not detected.

**Table 3:** Effect of biocontrol and non-biocontrol on mycotoxins concentration.

Mycotoxin	Biocontrol Effect (*p*-Values)
AFB1	0.0224 *
AFG1	0.0843
AFG2	0.1418
OTA	0.5262
FB1	0.8507
FB2	0.7598
FB3	0.8198
T2	0.1760
NIV	0.5015
ZEN	0.9724
FUS-X	0.4073
STE	0.0770

Statistically significant outcome at 95% confidence level (*p* < 0.05) is shown with *.

**Table 4:** Maize samples collected from study location (Aflasafe^®^ TZ01 treated and control).

Region	Treated (T)	Control (C)	Total
Chemba	38	38	76
Kiteto	41	41	82
Total	79	79	158

## Data Availability

The original contributions presented in this study are included in the article/Supplementary Material. Further inquiries can be directed to the corresponding author(s).

## Supplementary Materials

The following supporting information can be downloaded at: https://www.mdpi.com/article/10.3390/toxins17080419/s1, Table S1: MS/MS parameters for determination of 22 mycotoxins and two internal standards; Table S2: Concentrations (μg/kg) of detected mycotoxins for both treated and control maize samples.
